# Understanding health care needs among Veterans with Parkinson's disease: A survey study

**DOI:** 10.3389/fneur.2022.924999

**Published:** 2022-08-11

**Authors:** Megan Feeney, John Duda, Amie Hiller, Jay Phillips, Christiana Evers, Nicole Yarab, Veronica Todaro, Lydia Rader, Sheera Rosenfeld

**Affiliations:** ^1^Parkinson's Foundation, New York, NY, United States; ^2^Parkinson's Disease Research, Education and Clinical Center of the Michael J. Crescenz VA Medical Center, Department of Neurology of the University of Pennsylvania, Philadelphia, PA, United States; ^3^Department of Neurology, School of Medicine, Oregon Health and Science University, Portland, OR, United States; ^4^Department of Neurology, VA Portland Health Care System, Portland, OR, United States

**Keywords:** Veterans, Parkinson's disease, comprehensive care, mental health, falls

## Abstract

Among Veterans, it is estimated that 110,000 are living with Parkinson's disease (PD) in the United States. Whether or not Veterans living with PD are enrolled in the Veterans Health Administration (VHA), they may require special considerations when it comes to their care. We administered a survey to Parkinson's Foundation constituents with PD who had previously reported their Veteran status. Our goal was to identify areas where intervention can lead to improved health outcomes for Veterans living with Parkinson's disease. We specifically wanted to examine 1) the proportion of our Veteran constituents receiving services through the VHA, 2) the comprehensive care services that were utilized by Veterans living with PD, and 3) self-reported mental health and mobility status. We also wanted to compare those receiving care within and outside the VHA to see where there may be areas for improvement. With a response rate of 29.8% we received surveys from 409 United States Veterans with PD. As expected, mental health (MH) concerns in the previous 12 months were common with 36.0% of Veterans reporting concerns. Only 22.1% of respondents received care through VHA. Respondents with more falls and mental health concerns as well as those with higher levels of education and younger age were more likely to be seen at a VHA facility. In this sample, education level, household income, marital status, and VHA status were positively associated with increased health care utilization among Veterans. Those seen within the VHA were more likely to utilize MH and speech and language pathology consultation. This study highlights the importance of targeting educational outreach about care best practices for Veterans living with PD beyond VHA's current reach as well as the importance of access to good MH resources.

## Introduction

It is estimated that there are 19.16 million Veterans living in the United States, but less than half of those Veterans (9.16 million) are receiving services through the Veterans Health Administration (VHA) (1, 2). The VHA is the agency within the Department of Veterans Affairs (VA) that implements its healthcare programs. Among Veterans, it is estimated that 110,000 are living with Parkinson's disease (PD) in the United States (3, 4).

A Veteran living with Parkinson's disease and receiving VHA services may receive medical care at:

(1) A specialized Parkinson's Disease Research, Education, and Clinical Center (PADRECC). There are six PADRECC centers located in Philadelphia, Richmond, Houston, West Los Angeles, San Francisco, and Portland/Seattle (5, 6).
(2) A national VA Parkinson's Disease Consortium Network. These consortium networks have an affiliation with one of the six PADRECC sites and include approximately 55 more VHA sites with healthcare providers interested in improving care for Veterans with PD.
(3) A local VHA clinic that is not affiliated with a PADRECC site.

The PADRECCs aim to improve the quality of care and health outcomes for Veterans living with PD (5–7). The PADRECCs have movement disorder specialists on staff and focus on multidisciplinary care for PD, generally including specialized allied health services (5–7). PADRECCs also offer educational outreach for healthcare providers, Veterans living with PD, and care partners (5–7). Veterans living with PD were more likely to utilize these educational and support services when seen at a PADRECC (8). All the PADRECCs and Consortium sites also provide telemedicine services to Veterans who have difficulty getting to the sites (3).

Parkinson's disease is a neurodegenerative disorder that requires specialty and comprehensive medical treatment and care (9). Although there are successful treatments to help manage some PD symptoms (10), there is no cure for PD. Among Veterans, PD has a more negative impact on physical functioning and mental health than other chronic health conditions (11). Previous research specific to Veterans living with Parkinson's disease has primarily focused on the role of traumatic brain injury on the development or worsening of PD and PD symptoms (12–14), the associations between PD and mental health conditions such as depression and anxiety (15–17) the negative impacts of PD on quality of life (11, 18), and prodromal symptoms pf PD (19).

Whether or not Veterans living with PD are enrolled in the VHA, they require special considerations when it comes to their care particularly as it relates to mobility and mental health (11, 17). In this study, we administered a survey to Parkinson's Foundation constituents living with PD who had previously reported their Veteran status. Our primary purpose for this study was to identify where intervention(s) could lead to improved health outcomes for Veterans living with PD. To accomplish this, we had three goals when deploying this survey: 1) To understand the proportion of our Veteran constituents receiving services through the VHA, including PADRECCs and Consortium specialty centers, 2) To identify which comprehensive services were utilized by Veterans including movement disorder specialist care, physical and occupational therapy, speech and language pathology and mental health care, and 3) to understand self-reported mental health and mobility status. Due to the focus on comprehensive PD care and education, we expected that Veterans receiving health services at the VHA would have a higher utilization of specialty and comprehensive care services.

## Methods

### Standard protocol approvals, registrations, and patient consents

The Advarra institutional review board (IRB) approved this study. Given the minimal risk to participants, the Advarra IRB approved a waiver of written consent. A protocol-specific information sheet about the study which included the Informed Consent Form and Authorization to Use and Disclose Protected Health Information was electronically presented to respondents prior to starting the survey. Study participants had the option of completing the questionnaire anonymously or including their name and email if they wanted to be contacted for future studies.

### Participants

We invited two separate cohorts to complete an online survey; the first cohort consisted of Parkinson's Foundation constituents who had attended an online Parkinson's Foundation educational event for Veterans or were identified as a Veteran in our constituent database (*n* = 1,351), and the second cohort consisted of constituents recruited from various social media channels. People who reported a diagnosis of Parkinson's disease and identified as a Veteran of the United States military were eligible to complete the survey. Potential study participants received an initial email invitation and two additional weekly reminder emails. The initial invitation was sent July 21, 2021, and the survey remained open until August 20, 2021.

### Questionnaire

A review of publicly available surveys related to Veteran health and Parkinson92s disease was conducted, and relevant questions were used or modified to fit this survey (20–22). The survey consisted of eight sections (of which, seven were included in this manuscript). The survey was then reviewed by three Veterans with PD to ensure relevance and readability. The complete questionnaire is available in Supplementary Methods.

The online questionnaire consisted of eight sections:

1. Screening and demographic questions including where they receive healthcare services (*n* = 13).
2. Allied health referral questions for physical, occupational, and mental health therapy and speech and language pathology (*n* =16).
3. Mental health questions including concerns, reporting and assistance (*n* = 8).
4. Falls questions including near falls and reporting (*n* =5).
5. Telehealth questions related to utilization and satisfaction (*n* = 6).
6. Care partner involvement questions (*n* = 7).
7. *Tinetti Falls Efficacy Scale (FES) (n* = *10)* (21).
8. *Geriatric Depression Scale (GDS) (n* = *15)* (22).

Upon completion of the first six sections of the survey, respondents were asked if they would like to complete an additional survey the following week related to falls or mental health. If they selected yes and provided the Foundation with their email address, the FES and GDS were sent in the following 2 weeks.

There were two inclusion criteria for this study. Respondents needed to identify as a person with Parkinson's disease and a Veteran of the United States military. If respondents did not identify with both, they were excluded from the study. Of the 457 surveys returned from both cohorts combined, 403 came from the email cohort (29.83% response rate) and 54 came from the social media cohort. 38 respondents had not completed at least the demographics portion of the survey, and 10 did not meet inclusion criteria and were discarded from further analysis. There were 409 completed responses available for analysis (Supplementary Figure 1).

### Statistical analyses

The survey was collected and managed using Research Electronic Data Capture (REDCap) tools hosted at the Parkinson's Foundation (23, 24). Statistical analysis was performed using the R software package and programming language through RStudio desktop version 1.2.5042 (25, 26). Descriptive statistics were used to summarize survey responses (frequency and percentage for categorical variables, mean and standard deviation (SD) for continuous variables). Determination of associations between age, disease duration and age at diagnosis were performed using Student's *t*-test for continuous variables. Linear regression was performed to identify any associations with FES and GDS scores, considering demographics, provider types, referrals, or other health outcomes. Logistic regression was performed to compare demographics, provider types, and self-reported health outcomes between Veterans receiving care at the VHA and receiving care elsewhere. We also used logistic regression to compare allied health referrals, healthcare utilization, falls and mental health assistance. *P* < 0.05 was considered statistically significant. All tests were two-sided.

### Regression classifications

In our regression models: gender was sorted into two groups, male and female; household income was sorted into four groups, low income (< $50,000), middle income ($50,000–$149,999), high income (≥ $150,000) and prefer not to answer (27); education was sorted into three groups, less than a bachelor's degree, a bachelor's degree and a graduate degree; marital status was sorted into two groups, married and not married; type of doctor seen for PD was sorted into three groups, movement disorder specialist, neurologist or another doctor; if a respondent identified that they were seen at the VHA their status was classified as “seen at VHA,” otherwise it was classified as “not seen at VHA”; mental health concerns were dichotomized into “yes” and “no,” a “yes” response was coded if a respondent reported that they had experienced mental health concerns in the previous 12 months; frequent falls and near frequent falls were identified if a respondent indicated that they had experienced a fall or near fall at a “monthly,” “weekly” or “daily” rate, as opposed to “never” or “rarely.”

## Results

### Veteran demographics

We received surveys from 409 Veterans with self-reported PD from 47 states. Respondents were primarily white (94.1%) and male (91.7%). Respondents had an average age of 74.0 years (±8.2) and had PD for an average of 7.2 years (±7.3). Women were on average 7.5 years younger than the men in this sample (67.2 (±10.4) vs. 74.7 (±7.7), *p*-value < 0.01). Most respondents were retired (84.6%), middle income (50.6%) and had a bachelor's degree or higher (69.7%). Additional demographic information about survey respondents can be found in Table 1.

**Table 1:** Characteristics of the study population.

Age, mean (SD)	74.0 (8.2)
Duration of disease, mean (SD) Male, *n* (%) Female, *n* (%)	7.2 (7.3) 375 (91.7%) 34 (8.3%)
Race	
White, *n* (%) African American, *n* (%) Asian, *n* (%) American Indian or Alaska Native, *n* (%) Native Hawaiian or Pacific Islander, *n* (%) Some other race, ethnicity, or origin, *n* (%) Prefer not to answer, *n* (%)	385 (94.1%) 7 (1.7%) 5 (1.2%) 6 (1.5%) 0 (0.0%) 2 (0.5%) 9 (2.2%)
Ethnicity	
Hispanic, *n* (%)	10 (2.4%)
Education level	
Less than bachelor's degree, *n* (%) Bachelor's degree, *n* (%) Graduate degree, *n* (%)	124 (30.3%) 116 (28.4%) 169 (41.3%)
Household income	
Low (< $50,000), *n* (%) Middle ($50,000–$149,999), *n* (%) High (≥ $150,000), *n* (%) Prefer not to answer, *n* (%)	82 (20.0%) 207 (50.6%) 41 (10.0%) 79 (19.3%)
Marital status	
Married or DP, *n* (%) Not married, *n* (%) Prefer not to answer, *n* (%)	354 (86.6%) 53 (13.0%) 2 (0.5%)
Employment	
Employed, *n* (%) Unable to work, *n* (%) Unemployed, *n* (%) Retired, *n* (%) Prefer not to answer, *n* (%)	18 (4.4%) 41 (10.0%) 2 (0.5%) 346 (84.6%) 2 (0.5%)
Total participation: 409	

### Aim 1: Utilization of Veteran health services

Just over one fifth of respondents (22.1%) reported that they received care through the VHA. Respondents with higher levels of education, younger age, more falls, and mental health concerns were more likely to have been seen at a VHA after accounting for differences in gender, marital status, household income (HHI) and disease duration (Graduate degree OR = 2.2, 95%CI 1.1 to 4.8; Age OR = 0.93, 95%CI 0.90 to 0.96; Frequent Fall OR = 2.2, 95%CI 1.2 to 4.2; and MH Concerns OR = 2.6, 95%CI 1.4 to 4.6) (Table 2). Of those seen at the VHA (87), almost half (44.8%) reported that they were seen at a VHA medical center that offered specialized care for PD (PADRECC or Consortium center). However, of those seen at the VHA (87), about a quarter (23.0%) did not know if their VHA offered specialized care for PD. Due to a small sample size, we did not differentiate experiences between those who reported being seen at a special center for PD and those that did not or did not know.

**Table 2:** Adjusted analysis of individuals receiving services through the VHA.

**Seen at VHA**	**OR**	**(95% CI)**	**P**
(Intercept)	4.87	(0.38–61.13)	0.22
Gender: Male	2.96	(1.03–9.96)	0.06
Age	0.93	(0.90–0.96)	<0.01*
Education: Graduate degree	2.19	(1.05–4.78)	0.04*
Education: Less than bachelor's degree	1.39	(0.63–3.15)	0.42
Marital: Not married	1.76	(0.74–4.06)	0.19
Disease duration	1.03	(0.99–1.07)	0.08
Household income: Low-level	1.27	(0.41–4.08)	0.68
Household income: Mid-level	0.85	(0.33–2.39)	0.74
Household income: Prefer not to answer	0.93	(0.30–2.99)	0.90
Mental health concerns: Yes	2.56	(1.43–4.63)	<0.01*
Fall often: Yes	2.22	(1.16–4.24)	0.02*

The * symbol indicates the significant values at an alpha of 0.05.

### Aim 2: Utilization of comprehensive care services

Almost all respondents (90.5%) utilized at least one healthcare service in the previous 12 months *via* phone, virtual or in-person appointments (movement disorders specialist (MDS), primary care, allied health therapy, mental health and other services captured). The two healthcare providers most seen in the previous 12 months were primary care physicians (73.8%) and MDS (70.9%) (See Table 3). The least utilized service in the previous 12 months was occupational therapy (19.4%).

**Table 3:** Veteran self-reported utilization of healthcare service in the previous 12 Months.

	**Phone**	**Virtual**	**Person**	**None**	**N**
**MDS**	44 (12.94%)	102 (30.00%)	139 (40.88%)	99 (29.12%)	340
**General neuro**	13 (3.82%)	33 (9.71%)	76 (22.35%)	223 (65.59%)	340
**Mental health**	22 (6.47%)	39 (11.47%)	32 (9.41%)	247 (72.65%)	340
**PT**	9 (2.65%)	22 (6.47%)	123 (36.18%)	191 (56.18%)	340
**SLP**	7 (2.06%)	22 (6.47%)	53 (15.59%)	255 (75.00%)	340
**OT**	6 (1.76%)	11 (3.24%)	45 (13.24%)	274 (80.59%)	340
**PCP**	63 (18.53%)	65 (19.12%)	171 (50.29%)	89 (26.18%)	340
**Other**	33 (9.71%)	52 (15.29%)	115 (33.82%)	160 (47.06%)	340

+ Options were select all that apply in the previous 12 months, therefor percentages may add up to more than 100%.

The majority (76.9%) of Veteran respondents reported seeing a neurologist who specializes in Parkinson's (MDS) for treatment of their PD. Among all Veteran respondents, those with a graduate degree were more likely to see an MDS in the previous 12 months than those with a Bachelor's degree, whereas those with a low HHI were less likely to see an MDS in the previous 12 months than those with a high HHI after accounting for differences in gender, age, marital status, and VHA status (seen at the VHA or not) (Graduate degree OR = 2.3, 95% CI 1.3 to 4.3; Low HHI OR = 0.3, 95% CI 0.1 to 0.9).

Respondents with a physical therapy referral were more likely to have seen a physical therapist in the previous 12 months than those without a referral after accounting for differences in gender, age, education, marital status, disease duration, HHI, frequent falls and VHA status (OR = 9.96, 95% CI 5.17 to 20.73). When looking specifically at respondents with a physical therapy referral (216), those receiving services at the VHA were more likely to see a physical therapist at a 90% confidence level (*p* = 0.06).

Respondents with a mental health referral or mental health concerns were more likely to utilize mental health services in the previous 12 months (MH referral OR = 15.9 95% CI 6.4 to 43.8; MH concerns OR = 9.2, 95% CI 4.4 to 20.5), although respondents with less than a bachelor's degree, those not married, those with a middle HHI, and those seen at the VHA were also all associated with a higher likelihood of utilizing mental health services (Less than Bachelor's OR = 3.6 95% CI 1.3 to 10.3; Not married OR = 2.9, 95% CI 1.0 to 8.1; Middle HHI OR = 5.8 95% CI 1.2 to 44.9; Seen at VHA OR = 3.3, 95% CI 1.4 to 8.1). Respondents with a longer disease duration were less likely to utilize mental health services (OR = 0.93, 95% CI 0.86 to 0.99).

Respondents receiving services at the VHA and those with a middle HHI were more likely to use speech and language pathology (SLP) services in the previous 12 months than those not seen at the VHA or with a high HHI (Seen at VHA OR = 3.5, 95% CI 1.8 to 6.7; Middle HHI OR = 3.8, 95% CI 1.31 to 14.11), whereas those who were not married were less likely to utilize SLP services (OR = 0.3, 95% CI 0.1 to 0.8).

### Allied health referrals

Most respondents (75.1%) reported receiving at least one allied health or mental health referral from their PD provider (physical therapy, occupational therapy, speech language pathology or mental health therapy) since their diagnosis (See Table 4). The most common referral was for physical therapy (66.7%). The least common referral was for mental health services (14.4%). Those reporting mental health concerns and those receiving care at the VHA were more likely to receive a mental health referral than respondents not reporting concerns or receiving care elsewhere after accounting for differences in gender, age, education, marital status, disease duration, HHI, and type of doctor seen (MH concerns OR = 4.2 95% CI 2.1 to 8.6; Seen at VHA OR = 4.3, 95% CI 2.1 to 8.8).

**Table 4:** Referral and utilization rates for Veterans receiving Services at the VHA and elsewhere.

	**All respondents**		**VHA**		**Non-VHA**	
	**Referral (390)**	**Utilization (340)**	**Referral (87)**	**Utilization (74)**	**Referral (303)**	**Utilization (262)**
Physical therapy	260 (66.7%)	143 (42.1%)	57 (65.5%)	37 (50.0%)	202 (66.7%)	106 (40.4%)
Occupational therapy	111 (28.5%)	57 (16.8%)	25 (28.7%)	16 (21.6%)	85 (28.1%)	41 (15.6%)
Speech and language pathology^a^	161 (41.3%)	76 (22.4%)	42 (48.3%)	29 (39.2%)	118 (38.9%)	47 (17.9%)
Mental health therapy^b^	56 (14.4%)	81 (23.8%)	29 (33.3%)	36 (48.6%)	27 (8.9%)	45 (17.2%)

a Inregression model, VHA significantly associated with utilization (Seen at VHA OR = 3.5, 95% CI 1.8 to 6.7).

b In regression model, VHA significantly associated with referral (Seen at VHA OR = 4.3, 95% CI 2.1 to 8.8) and utilization (Seen at VHA OR = 3.3, 95% CI 1.4 to 8.1).

### Aim 3: Self-reported mental health and mobility

#### Self-reported mental health status

Most respondents (74.3%) rated themselves in good, very good or excellent mental health. However, more than a third of respondents (35.9%) reported mental health concerns in the previous 12 months. Respondents seen at the VHA were more likely to rate themselves in poorer mental health (*p*-value < 0.001) and more likely to report mental health concerns in the previous 12 months (*p*-value < 0.001). Respondents discussed many mental health topics with their provider including problems with sleep (80.8%), depression or feeling down (68.9%), anxiety or nervousness (62.2%), hallucinations (seeing or hearing something that isn't real or there) (53.2%), apathy or a lack of interest (47.0%), obsessions and compulsions (difficulty controlling your impulses like compulsive gambling, eating or sex) (31.9%), and post-traumatic stress disorder (PTSD) (17.8%). A small minority of respondents (10.5%) shared that they had not discussed any of the above mental health issues with a provider.

Respondents were asked to whom they reported their mental health concerns. Many respondents reported these concerns to family (60.8%), their doctors (PD doctor 60.0%, PCP 52.2%), but not their friends (31.9%). About a quarter (24.1%) of respondents did not discuss their mental health concerns to any of the options provided. Respondents receiving care at the VHA compared to those receiving care elsewhere were more likely to discuss their concerns with someone when accounting for differences in age, education, marital status, disease duration, HHI, and type of PD doctor seen (OR = 3.7, 95% CI 1.5 to 11.2).

Most respondents (75.7%) also reported knowing where to go for mental health assistance. Respondents receiving services at the VHA were more likely to know where to get assistance than those not receiving services at the VHA (OR = 3.6, 95% CI 1.5 to 10.2), whereas men were less likely to know where to get mental health assistance than women after accounting for differences in age, education, marital status, disease duration, HHI, type of PD doctor seen, and mental health concerns (OR = 0.01, 95% CI 0.01 to 0.57).

**Figure 1:**
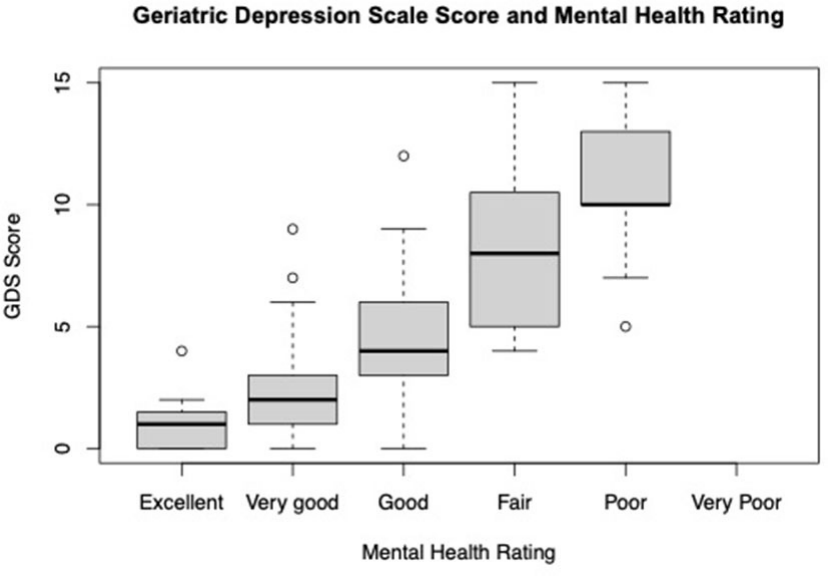
Average GDS score by self-reported mental health rating (*n* = 105).

A subset of respondents (28.4%) agreed to take the Geriatric Depression Scale (GDS) one week following the original survey. The average GDS score among respondents was 5.12 (±3.9). According to GDS scoring guidelines, almost half (45.7%) of this subset of respondents reported scores suggestive of mild, moderate, or severe depression (23.8%, 13.3%, and 8.6% respectively). There were not self-reported mental health rating differences (excellent, very good, good, fair, poor, and very poor) between those that completed the GDS and those that did not. Linear regression showed GDS scores to be positively associated with respondents self-reported mental health rating of good, fair, or poor compared to excellent after accounting for differences in gender, age, education, marital status, disease duration, HHI, type of PD doctor seen, VHA status and mental health concerns (Mental Health ratings: Good *p*-value < 0.01, Fair *p*-value < 0.01, Poor *p*-value < 0.01) (Figure 1).

### Self-reported falls and near falls

About one-fifth of respondents (21.3%) reported frequent falls (daily, weekly, or monthly). A little less than half of respondents (41.8%) reported frequent near falls (Supplementary Table 1). Of the respondents falling often, nearly one fifth (17.9%) had not yet received a PT referral. Respondents receiving care at the VHA were more likely to fall often than those receiving care elsewhere (OR = 2.3, 95% CI 1.1 to 5.2) and respondents frequently experiencing near falls were more likely to frequently fall than those not experiencing frequent near falls (OR = 40.4, 95% CI 15.4 to 139.9), when accounting for differences in gender, age, education, marital status, disease duration, HHI, type of doctor seen, and PT referral status. Respondents did not always report falls to PD physicians (61.7% reported), physical therapists (33.6% reported) or care partners (72.5% reported). Nearly one fifth of respondents (19.3%) that experienced a fall did not report it to anyone.

**Figure 2:**
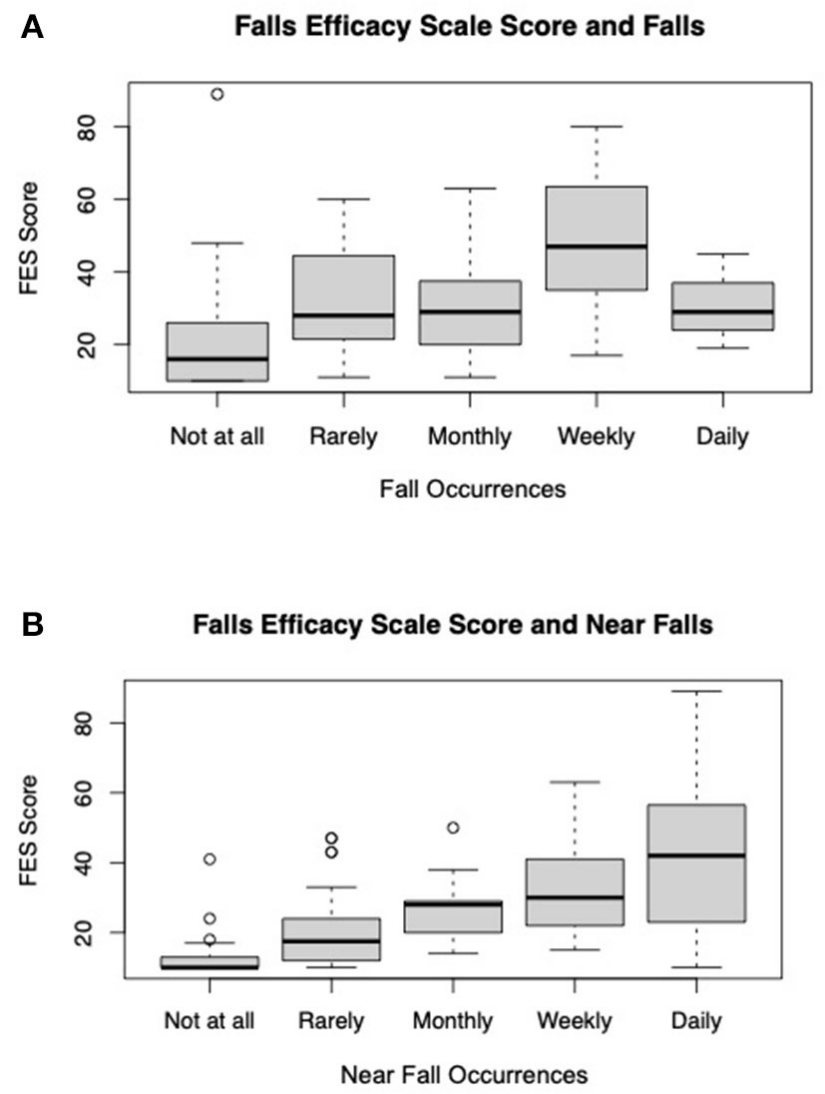
Average FES score by **(A)** average FES score by self-reported falls and **(B)** average FES Score by self-reported near falls (*n* = 115).

A subset of respondents (115) agreed to take the Tinetti Falls Efficacy Scale (FES) 1 week following the original survey. An FES score above 70 indicates that the respondent has a fear of falling. There were not reported fall frequency differences between those that completed the FES and those that did not. The average FES score was 26.8 (±17.23). Linear regression showed FES scores to be positively associated with respondents self-reported falls (weekly *p*-value < 0.01), and near falls (monthly *p*-value = 0.03, weekly *p*-value = 0.01, and daily *p*-value < 0.01) after accounting for differences in gender, age, education, marital status, disease duration, HHI, type of PD doctor seen, VHA status, and PT referral status and (Figure 2).

## Discussion

The results of this study describe 1) the proportion of our Veteran constituents receiving services through the VHA, 2) the comprehensive care services that were utilized by Veterans living with PD, and 3) self-reported mental health and mobility status. This study also highlighted differences between Veterans seen at the VHA and Veterans seen elsewhere. Our findings suggested that about one fifth of Veteran respondents received medical services through the VHA, while the remaining four-fifths received medical care elsewhere. Almost all Veteran respondents had utilized at least one care service in the previous 12 months, but there were utilization differences between Veterans receiving services at the VHA and those receiving services elsewhere. Veterans receiving health services at the VHA had a higher utilization of some specialty and comprehensive care services. Diving further into this, we also found referral differences between the two groups. Lastly, mental health concerns and frequent falls were more likely to be reported by Veterans receiving services at the VHA than those receiving services elsewhere.

In our cohort, just over one fifth of respondents received medical services through the VHA. This is slightly below the estimated percentage among all Veterans (not specific to PD) utilizing VHA services (27.3%) (28, 29). Among our respondents, those seen at the VHA had higher self-reported incidence of falls and mental health concerns. Based upon these findings, we have identified targeted recommendations to further improve the care and health outcomes of Veterans living with PD (Table 5). Veterans seen at the VHA in this sample reported a more negative health status. This is consistent with other research that found that Veterans seen at the VHA had a lower physical and mental quality of life than veterans receiving services elsewhere (30). Although our survey did not find socio-economic status to be associated with VHA status, previous research has shown that Veterans receiving services through the VHA were more likely to have a lower SES (31–33). This discrepancy may be an artifact of the reality that Veterans with higher SES are more likely to have access to the internet and to be aware of the Parkinson's Foundation. It may also relate to the high prevalence of service connection among veterans with PD (persons with higher socio-economic status do not generally qualify to receive VA care unless they are service connected).

**Table 5:** Recommendations for improving health utilization and outcomes of Veterans.

**Type of finding**	**Findings**	**Recommendations**
Utilization of veteran health services, allied health referrals, and other health services	•Veterans seen at the VHA had higher self-reported incidence of falls and mental health problems •Veterans seen at the VHA were more likely to receive referrals and utilize health services	•Veterans at the VHA may be in greater need of mental health and physical therapy referrals •Future studies should compare referrals and utilization at VHA Parkinson's disease specialty centers, VHA non-specialty centers, and care received in the community •Protocols for equitable care for veterans outside of the VHA are needed
Self-reported mental health status	•About 25% of respondents reported fair or poor mental health •About half of respondents who took the GDS reported some level of depression •Men and those seen outside of the VHA are less likely to know how to access mental health services •Respondents seen outside of the VHA were less likely to report their mental health concerns	•Early referrals to mental health services for Veterans are needed •Provide educational outreach to healthcare workers for best care practices for Veterans seen within and outside the VHA •Host community efforts that connect Veterans living with PD to Veterans care resources •Increase partnerships between organizations, such as between the Parkinson's Foundation and the VHA, to share resources and services
Self-reported falls and near falls	•Almost half of respondents reported frequent falls or near falls •Respondents frequently did not report falls to their PD physicians (61.7%) •Of respondents frequently falling, 17.9% have not yet received a PT referral	•When in person visits are not possible, providers should utilize telemedicine to assess about fall risks •Healthcare professionals should ask about both fall AND near falls at all PD evaluations •Future studies should compare Veteran fall risk to non-Veterans prospectively and try to identify modifiable causes

Recommendations based on the results of the current paper. GDS, Geriatric Depression Scale: MDS, Movement Disorder Specialist.

In this sample, education level, HHI, marital status, and VHA status were associated with care utilization among Veterans. Among Veterans not receiving services through the VHA, higher education was associated with higher reported rates of MDS care. Although not all Veterans with PD are eligible to receive care through the VHA, all Veterans can benefit from access to Parkinson's specialists. This study highlights the need for equitable care and allied health services for veterans receiving services outside of the VHA (Table 5).

Veterans receiving services through the VHA were also more likely to rate themselves in poorer mental health. As is well established in the literature, more work is needed surrounding diagnosing and treating mental health among Veterans with PD (11, 17, 34, 35). This was particularly true for men and those seen outside the VHA, as these two groups were less likely to know how to access mental health assistance, and those receiving services outside of the VHA were less likely to discuss concerns with friends, family or medical and mental health providers.

Falls were common in our population and many persons did not report their falls to any of their providers. It is known that past falls predict future falls, falls can have a huge impact on quality of life, and are even associated with reduced life expectancy (36). To help prevent future fall-related negative health outcomes, medical providers should ask about both falls **and** near falls in medical appointments, as near falls were associated with frequent falls in this sample. These discussions could be particularly important at the VHA, as respondents in this sample receiving care through the VHA were more likely to experience frequent falls (Table 5).

Research among all Veterans (not specific to those living with PD) indicated that mental health issues were more prevalent among Veterans receiving services through the VHA (37). Early referrals to allied health and mental health services are key to maintaining quality of life for Veterans living with PD. This study highlighted the importance of referrals by demonstrating their positive association with later utilization (MH and PT). However, in this sample, MH referrals were more likely to be provided to Veterans receiving services at the VHA. Although the VHA has the goal of prioritizing comprehensive care, outreach about the importance of comprehensive care for Veterans living with PD is lacking among medical and allied health professionals serving Veterans outside of the VHA. In parallel with educating medical and allied health professionals, education efforts are needed to connect Veterans to eligible services. Many veterans may not know that they are able to receive services through the VHA, fully understand the availability of PD-specific care provided by the VHA, or the possibility of receiving benefits from the VA for certain service-related exposures associated with PD. Community efforts to reach and educate Veterans living with PD and share specific resources, like the Parkinson's Foundation partnership with the VHA and PADRECC networks as well as their national and regional community programs, can help direct Veterans to crucial resources and services (Table 5) (38).

As we expected, Veterans receiving health services at the VHA had a higher utilization of specialty and comprehensive care providers. Veterans receiving services at the VHA were more likely to receive referrals for MH and SLP and utilize MH services. Although the referral and utilization rates for PT and OT were similar for Veterans receiving services at the VHA and Veterans receiving services elsewhere, across all specialties, utilization was higher in the VHA group. Our findings suggest that that VHA's work to reduce inequities in care (39–43) have been successful in this cohort. Further investigation into the causes of utilization differences may yield opportunities to improve utilization among Veterans not receiving services at the VHA (cost of therapy, accessibility of providers, knowledge about the role of PT/OT in PD care, etc.). Due to a small sample size, we were unable to compare VHA PADRECCs and non-specialty centers, however, future studies should consider comparing referrals and utilization rates among these two types of centers (Table 5).

There are several limitations to this study. Respondents were acquired through a convenience sample, and as a result the demographics reflected in this study primarily consisted of married and highly educated respondents, which likely does not reflect the entire population of Veterans living with PD. As this was a convenience sample, self-selection may have been a source of bias in this sample. The study response rate of 29.83% also suggests that respondents may not have been representative of the Veterans living with PD population. Although our sample was <500, our respondent demographics were similar to previous research studies hosted by the Foundation (20, 44, 45). All data in this study were self-reported. All respondents were required to complete the survey electronically, and this survey invitation was sent only to those who had an active email address or had a social media account (Facebook). Given that PD predominantly affects the older population, it is possible that the technology and email requirements excluded older Veterans who did not have an active email address, access to an electronic or mobile device or access to the internet. This was a cross-sectional study so there are possible instances of reverse causality in some relationships. In addition, this study, which was not longitudinal, cannot demonstrate if a population is improving with appropriate resources/referrals in place.

Although this survey was deployed during the coronavirus pandemic, our findings related to utilization of services was not different from other surveys we pushed in this same time frame (20, 44). Although not reported in detail here, a portion of this survey was dedicated to telehealth services. The care utilization rate of 90.5% included in-person and telehealth appointments. Although the coronavirus pandemic has affected many aspects of healthcare, due to the high utilization rates in our study, we do not believe that Veteran care was impacted differently from the overall population. This may be due to the VHA having an established telehealth network prior to the start of pandemic.

In summary, a large survey of Veterans living with PD was conducted to summarize care utilization of the health services and self-reported health status. This survey also sought to identify care differences between Veterans seen at the VHA and Veterans seen elsewhere. In this study, Veterans receiving services at the VHA were more likely to report a negative health status, and they were more likely to receive and utilize care for specific allied and mental health services. This study highlighted the importance of targeting educational outreach about care best practices for Veterans living with PD beyond VHA's current reach, including healthcare professionals outside of the VHA. Partnerships between the VHA and organizations like, Parkinson's Foundation, can help guide eligible Veterans to the VHA and assist non-eligible Veterans in finding specialized care and services.

## Data availability statement

The data that support the findings of this study are available from the corresponding author SR, upon reasonable request.

## Ethics statement

The studies involving human participants were reviewed and approved by Advarra Institutional Review Board (IRB). Written informed consent for participation was not required for this study in accordance with the national legislation and the institutional requirements.

## Author contributions

MF: research project organization and execution, survey design, execution and review of statistical analysis, and writing of first draft of the manuscript. JP, VT, CE, NY, and SR: survey design and review of manuscript. LR, AH, and JD: review of manuscript. All authors contributed to the article and approved the submitted version.

## Funding

This work was self-funded by Parkinson's Foundation.

## Conflict of interest

The authors declare that the research was conducted in the absence of any commercial or financial relationships that could be construed as a potential conflict of interest.

## Publisher's note

All claims expressed in this article are solely those of the authors and do not necessarily represent those of their affiliated organizations, or those of the publisher, the editors and the reviewers. Any product that may be evaluated in this article, or claim that may be made by its manufacturer, is not guaranteed or endorsed by the publisher.
