# Supplementary material for: Understanding health care needs among Veterans with Parkinson's disease: A survey study
Source: Front Neurol. 2022 Aug 11;13:924999. doi: 10.3389/fneur.2022.924999 (PMC9405651; doi:10.3389/fneur.2022.924999)
Supplement: Supplementary file 1 [file Data_Sheet_1.PDF]

PARKINSON’S FOUNDATION VETERANS SURVEY

Survey Link:

Variable / Field Name	Form Name	Section Header	Field Type	Field Label	Choices, Calculations, OR Slider Labels	Branching Logic (Show field only if...)	Required Field?
record_id	veterans_survey_demographics		text	Record ID			
info_sheet	veterans_survey_demographics		descriptive	For more information about this study, please review the sheet here:			
screen_pwp	veterans_survey_demographics		radio	Have you received a Parkinson's disease diagnosis from a doctor or health care professional?	1, Yes | 2, No		y
screen_vet	veterans_survey_demographics		radio	Are you a veteran of the United States military?	1, Yes | 2, No		y

birth_year	veterans_survey_demographics	Demographics	dropdown	In what year were you born?	1916, 1916 | 1917, 1917 | 1918, 1918 | 1919, 1919 | 1920, 1920 | 1921, 1921 | 1922, 1922 | 1923, 1923 | 1924, 1924 | 1925, 1925 | 1926, 1926 | 1927, 1927 | 1928, 1928 | 1929, 1929 | 1930, 1930 | 1931, 1931 | 1932, 1932 | 1933, 1933 | 1934, 1934 | 1935, 1935 | 1936, 1936 | 1937, 1937 | 1938, 1938 | 1939, 1939 | 1940, 1940 | 1941, 1941 | 1942, 1942 | 1943, 1943 | 1944, 1944 | 1945, 1945 | 1946, 1946 | 1947, 1947 | 1948, 1948 | 1949, 1949 | 1950, 1950 | 1951, 1951 | 1952, 1952 | 1953, 1953 | 1954, 1954 | 1955, 1955 | 1956, 1956 | 1957, 1957 | 1958, 1958 | 1959, 1959 | 1960, 1960 | 1961, 1961 | 1962, 1962 | 1963, 1963 | 1964, 1964 | 1965, 1965 | 1966, 1966 | 1967, 1967 | 1968, 1968 | 1969, 1969 | 1970, 1970 | 1971, 1971 | 1972, 1972 | 1973, 1973 | 1974, 1974 | 1975, 1975 | 1976, 1976 | 1977, 1977 | 1978, 1978 | 1979, 1979 | 1980, 1980 | 1981, 1981 | 1982, 1982 | 1983, 1983 | 1984, 1984 | 1985, 1985 | 1986, 1986 | 1987, 1987 | 1988, 1988 | 1989, 1989 | 1990, 1990 | 1991, 1991 | 1992, 1992 | 1993, 1993 | 1994, 1994 | 1995, 1995 | 1996, 1996 | 1997, 1997 | 1998, 1998 | 1999, 1999 | 888, Prefer not to answer		y

dx_year	veterans_survey_demographics		dropdown	In what year were you diagnosed with Parkinson's disease	1951, 1951 | 1952, 1952 | 1953, 1953 | 1954, 1954 | 1955, 1955 | 1956, 1956 | 1957, 1957 | 1958, 1958 | 1959, 1959 | 1960, 1960 | 1961, 1961 | 1962, 1962 | 1963, 1963 | 1964, 1964 | 1965, 1965 | 1966, 1966 | 1967, 1967 | 1968, 1968 | 1969, 1969 | 1970, 1970 | 1971, 1971 | 1972, 1972 | 1973, 1973 | 1974, 1974 | 1975, 1975 | 1976, 1976 | 1977, 1977 | 1978, 1978 | 1979, 1979 | 1980, 1980 | 1981, 1981 | 1982, 1982 | 1983, 1983 | 1984, 1984 | 1985, 1985 | 1986, 1986 | 1987, 1987 | 1988, 1988 | 1989, 1989 | 1990, 1990 | 1991, 1991 | 1992, 1992 | 1993, 1993 | 1994, 1994 | 1995, 1995 | 1996, 1996 | 1997, 1997 | 1998, 1998 | 1999, 1999 | 2000, 2000 | 2001, 2001 | 2002, 2002 | 2003, 2003 | 2004, 2004 | 2005, 2005 | 2006, 2006 | 2007, 2007 | 2008, 2008 | 2009, 2009 | 2010, 2010 | 2011, 2011 | 2012, 2012 | 2013, 2013 | 2014, 2014 | 2015, 2015 | 2016, 2016 | 2017, 2017 | 2018, 2018 | 2019, 2019 | 2020, 2020 | 2021, 2021 | 888, Prefer not to answer		y
marital	veterans_survey_demographics		radio	What is your marital status?	1, Single (never married) | 2, Married or domestic partnership | 3, Separated | 4, Widowed | 5, Divorced | 888, Prefer not to answer		y
gender	veterans_survey_demographics		radio	Please select the gender you identify with:	1, Male | 2, Female | 3, Other | 888, Prefer not to answer		y
race_ethnicity	veterans_survey_demographics		checkbox	Please select the categories that describe you: Please select all that apply.	AmInd, American Indian or Alaska Native | Asian, Asian | Black, Black/African American/African/Caribbean | Hispanic, Hispanic/Latino | Hawaiian, Native Hawaiian Pacific Islander | White, White | Other, Some other race, ethnicity or origin | 888, Prefer not to answer		y

employment	veterans_survey_demographics		radio	What is your current employment status?	1, Employed full time | 2, Employed part-time | 3, Self-employed | 4, Unemployed | 5, Retired | 6, Unable to work or disabled | 888, Prefer not to answer		y
education	veterans_survey_demographics		radio	What is your highest level of education?	1, Less than a high school diploma | 2, High school degree or equivalent (e.g. GED) | 3, Some college, no degree | 4, Trade/technical school (vocational training) | 5, Associate degree (e.g. AA, AS) | 6, Bachelor's degree (e.g. BA, BS) | 7, Master's degree (e.g. MA, MS, MEd) | 8, Professional degree (e.g. MD, DDS, DVM, JD) | 9, Doctorate (e.g. PhD, EdD) | 888, Prefer to not answer		y
income	veterans_survey_demographics		radio	What is your current yearly household income? Include income from Social Security, investment, etc.).	1, Less than $25,000 | 2, $25,000 to $34,999 | 3, $35,000 to $49,999 | 4, $50,000 to $74,999 | 5, $75,000 to $99,999 | 6, $100,000 to $149,999 | 7, $150,000 to $199,999 | 8, $200,000 or more | 888, Prefer not to answer		y
zip	veterans_survey_demographics		text	What is your current zip code?			y
covid_19	veterans_survey_demographics		radio	Since the beginning of the coronavirus pandemic, have you been diagnosed with COVID-19 by a doctor or healthcare professional?	1, Yes | 2, No | 888, Prefer not to answer		y
help_w_survey	veterans_survey_demographics		radio	Is anyone helping you complete this survey today?	1, Yes, my care partner | 2, Yes, a family member who is not my primary care partner | 3, Yes, a friend who is not my primary care partner | 4, No, I am completing the survey on my own | 888, Prefer not to answer		y

referral_provider	veterans_survey_referrals	These next few questions are about the doctor or medical provider you see most often for treatment related to your Parkinson's disease.	radio	What type of doctor or medical provider do you see most often for treatment related to your Parkinson's disease?	1, Primary care or family doctor | 2, Neurologist who specializes in Parkinson's (movement disorder specialist) | 3, General neurologist | 4, Nurse who specializes in Parkinson's | 5, Another health care professional (including a physician's assistant or general nurse practitioner) | 6, I don't have a doctor or medical provider treating me for my Parkinson's | 7, I don't know | 888, Prefer not to answer		y
referral_dx	veterans_survey_referrals		radio	Is this the doctor or medical provider that diagnosed you with Parkinson's disease?	1, Yes | 2, No | 888, Prefer not to answer	[referral_provider] = '1' or [referral_provider] = '2' or [referral_provider] = '3' or [referral_provider] = '4' or [referral_provider] = '5'	y
referral_location	veterans_survey_referrals		radio	Where do you see the doctor or medical provider for treatment related to your Parkinson's disease?	1, Veteran's Administration (VA) medical center | 2, University, hospital or hospital network that partners with the Veteran's Administration (VA) | 3, University medical center | 4, Hospital or hospital network | 5, Combination university and hospital or hospital network | 6, Health maintenance organization network (such as Kaiser Permanente) | 7, Group practice | 8, Private practice | 9, Community health clinic | 10, Other | 11, I don't know | 888, Prefer not to answer		y
referral_vacenter	veterans_survey_referrals		radio	Are you seen at a VA medical center that offers specialized care in Parkinson's disease? If you are unsure, please select "I don't know".	1, Yes | 2, No | 3, I don't know | 888, Prefer not to answer	[referral_location] = '1' or [referral_location] = '2'	y
referral_dbs	veterans_survey_referrals		radio	Have you received deep brain stimulation (DBS) as a treatment for Parkinson's disease symptoms?	1, Yes | 2, No | 888, Prefer not to answer		y

referral_pt	veterans_survey_referrals		radio	Since your diagnosis, has your doctor or medical provider referred you to a physical therapist?	1, Yes | 2, No | 3, I don't know | 888, Prefer not to answer	[referral_provider] = '1' or [referral_provider] = '2' or [referral_provider] = '3' or [referral_provider] = '4' or [referral_provider] = '5' or [referral_provider] = '7'	y
referral_pt_time	veterans_survey_referrals		radio	How soon after your diagnosis were you referred to a physical therapist?	1, Within one month | 2, Between two and six months | 3, Between six months and one year | 4, Between one and five years | 5, More than five years | 6, I don't recall | 888, Prefer not to answer	[referral_pt] = '1'	y
referral_pt_reason	veterans_survey_referrals		radio	For what reason was this referral provided?	1, I experienced a fall | 2, My provider was concerned about balance issues or a potential fall | 3, Some other reason | 4, I don't know | 888, Prefer not to answer	[referral_pt] = '1'	y
referral_ot	veterans_survey_referrals		radio	Since your diagnosis, has your doctor or medical provider referred you to an occupational therapist?	1, Yes | 2, No | 3, I don't know | 888, Prefer not to answer	[referral_provider] = '1' or [referral_provider] = '2' or [referral_provider] = '3' or [referral_provider] = '4' or [referral_provider] = '5' or [referral_provider] = '7'	y
referral_ot_time	veterans_survey_referrals		radio	How soon after your diagnosis were you referred to an occupational therapist?	1, Within one month | 2, Between two and six months | 3, Between six months and one year | 4, Between one and five years | 5, More than five years | 6, I don't recall | 888, Prefer not to answer	[referral_ot] = '1'	y

referral_slp	veterans_survey_referrals		radio	Since your diagnosis, has your doctor or medical provider referred you to a speech-language pathologist?	1, Yes | 2, No | 3, I don't know | 888, Prefer not to answer	[referral_provider] = '1' or [referral_provider] = '2' or [referral_provider] = '3' or [referral_provider] = '4' or [referral_provider] = '5' or [referral_provider] = '7'	y
referral_slp_time	veterans_survey_referrals		radio	How soon after your diagnosis were you referred to a speech-language pathologist?	1, Within one month | 2, Between two and six months | 3, Between six months and one year | 4, Between one and five years | 5, More than five years | 6, I don't recall | 888, Prefer not to answer	[referral_slp] = '1'	y
referral_mh	veterans_survey_referrals		radio	Since your diagnosis, has your doctor or medical provider referred you to a mental health provider? A mental health provider can include a psychiatrist, psychologist, psychiatric-mental health nurse, psychiatric physician assistant, licensed clinical social worker or a licensed professional counselor.	1, Yes | 2, No | 3, I don't know | 888, Prefer not to answer	[referral_provider] = '1' or [referral_provider] = '2' or [referral_provider] = '3' or [referral_provider] = '4' or [referral_provider] = '5' or [referral_provider] = '7'	y
referral_mh_time	veterans_survey_referrals		radio	How soon after your diagnosis were you referred to a mental health provider?	1, Within one month | 2, Between two and six months | 3, Between six months and one year | 4, Between one and five years | 5, More than five years | 6, I don't recall | 888, Prefer not to answer	[referral_mh] = '1'	y
referral_homehealth	veterans_survey_referrals		radio	Do you have professional home health assistance come to your home to provide care services for your Parkinson’s disease? Home health services can include skilled nursing care, occupational therapy, physical therapy, durable medical equipment, and other personal healthcare-related services.	1, Yes, through the VA | 2, Yes, through another service | 3, No | 4, I don't know | 888, Prefer not to answer		y

referral_homehealth_type	veterans_survey_referrals		radio	For what services do you use home health assistance for your Parkinson's disease?	1, Part-time or "intermittent" skilled nursing care | 2, Physical therapy | 3, Occupational therapy | 4, Speech-language pathology services | 5, Medical social services | 6, Part-time or intermittent home health aide services (personal hands-on care) | 888, Prefer not to answer	[referral_homehealth] = '1' or [referral_homehealth] = '2'	y
mh_topics_discussed	veterans_survey_mental_health	The next section will ask questions related to your mental health. Mental health can include depression, anxiety, loss of motivation, stress, problems with emotions, psychosis, cognition, compulsive behaviors, or post-traumatic stress disorder at one point in time or over an extended period of time.	checkbox	Since your diagnosis, has your doctor or medical provider ever asked you whether you experience any of the following: Please select all that apply.	1, Problems with sleep | 2, Anxiety or nervousness | 3, Depression or feeling down | 4, Apathy or a lack of interest | 5, Post-traumatic stress disorder (PTSD) | 6, Hallucinations (seeing or hearing something that isn't real or there) | 7, Obsessions and compulsions (difficulty controlling your impulses like compulsive gambling, eating or sex) | 8, None of the above | 888, Prefer not to answer		y
mh_concerns	veterans_survey_mental_health		radio	In the past 12 months, have you had concerns about your mental health?	1, Yes | 2, No | 888, Prefer not to answer		y
mh_rating	veterans_survey_mental_health		radio	In general, how would you rate your mental health?	1, Excellent | 2, Very good | 3, Good | 4, Fair | 5, Poor | 6, Very poor | 888, Prefer not to answer		y
mh_change	veterans_survey_mental_health		radio	How do you think that your mental health has changed since your Parkinson's diagnosis?	1, Much improved | 2, Slightly improved | 3, No change | 4, Slightly worse | 5, Much worse | 888, Prefer not to answer		y
mh_talk_family	veterans_survey_mental_health	Have you discussed mental health concerns with any of the following:	radio	Family	1, Yes | 2, No | 888, Prefer not to answer		y
mh_talk_friends	veterans_survey_mental_health		radio	Friends	1, Yes | 2, No | 888, Prefer not to answer		y
mh_talk_pd_doctor	veterans_survey_mental_health		radio	The doctor or medical provider who treats you for your Parkinson's	1, Yes | 2, No | 888, Prefer not to answer		y

mh_talk_doctor	veterans_survey_mental_health		radio	Your primary care physician or family doctor	1, Yes | 2, No | 888, Prefer not to answer		y
mh_talk_mh	veterans_survey_mental_health		radio	A mental health professional	1, Yes | 2, No | 888, Prefer not to answer		y
mh_talk_why	veterans_survey_mental_health		notes	Why have you not discussed mental health concerns with your provider?		[mh_talk_pd_doctor] = '2' or [mh_talk_doctor] = '2'	y
mh_help	veterans_survey_mental_health		radio	Do you know where to reach out for assistance for mental health concerns?	1, Yes | 2, No | 3, I don't know | 888, Prefer not to answer		y
mh_help_open	veterans_survey_mental_health		notes	Where would you reach out for assistance for mental health concerns?		[mh_help] = '1'	y
falls_fall	veterans_survey_falls		radio	In the last month, how frequently have you fallen down? By falling down, we mean any fall, slip, or trip in which you lose your balance and land on the floor or ground or at a lower level.	1, Not at all | 2, Rarely | 3, Monthly | 4, Weekly | 5, Daily | 888, Prefer not to answer		y
falls_reported	veterans_survey_falls		checkbox	To whom were these falls reported?Please select all that apply.	1, Your care partner | 2, The doctor or medical provider you see for your Parkinson's disease | 3, Your physical therapist | 4, No one | 888, Prefer not to answer	[falls_fall] = '2' or [falls_fall] = '3' or [falls_fall] = '4' or [falls_fall] = '5'	y
falls_near_fall	veterans_survey_falls		radio	In the last month, how frequently have you experienced a near fall? A near fall is an instance in which you started to fall but caught yourself.	1, Not at all | 2, Rarely | 3, Monthly | 4, Weekly | 5, Daily | 888, Prefer not to answer		y
falls_nearfall_report	veterans_survey_falls		checkbox	To whom were these near falls reported?	1, Your care partner | 2, The doctor or medical provider you see for your Parkinson's disease | 3, Your physical therapist | 4, No one | 888, Prefer not to answer	[falls_near_fall] = '2' or [falls_near_fall] = '3' or [falls_near_fall] = '4' or [falls_near_fall] = '5'	y
falls_limited_activity	veterans_survey_falls		checkbox	Do you limit your activity (such as walking) to prevent falls? For example, do you limit walking to when you can hold on to something or someone?	1, Yes | 2, No | 888, Prefer not to answer		y

tele_use	veterans_survey_telehealth	The next set of questions will ask you about your experiences with telehealth or virtual appointments for your Parkinson's disease conducted over the phone or video.	radio	In the past 12 months, have you received care or consultations via telehealth or a virtual appointment?	1, Yes | 2, No | 3, I don't know | 888, Prefer not to answer		
tele_no_use	veterans_survey_telehealth		checkbox	For what reason(s) have you not participated in telehealth in the past 12 months? Please select all that apply.	1, It was not offered to me | 2, I did not need to see a doctor or other health care provider in the past year | 3, I was not able to get a telehealth appointment with my regular doctor or health care provider | 4, I could not figure out how to schedule a telehealth appointment | 5, I could not figure out how to log into a telehealth appointment | 6, I have no interest in using telehealth services | 7, I did not feel that it would be as thorough as an in-person appointment | 8, My doctor was hesitant to engage in telehealth appointments | 9, I do not have an electronic device (computer, tablet, smartphone) that supports telehealth appointments | 10, I do not have reliable internet | 11, Other | 888, Prefer not to answer	[tele_use] = '2'	y
tele_doc_pcp	veterans_survey_telehealth	In the past 12 months, in what types of telehealth appointments did you participate? Please select all that apply.	checkbox	A primary care or family doctor or provider	1, Appointment by phone | 2, Appointment by video | 3, Appointment in person | 4, No appointment with this health professional | 888, Prefer not to answer		y
tele_doc_mds	veterans_survey_telehealth		checkbox	A neurologist who specializes in Parkinson's (movement disorder specialist)	1, Appointment by phone | 2, Appointment by video | 3, Appointment in person | 4, No appointment with this health professional | 888, Prefer not to answer		y

tele_doc_neuro	veterans_survey_telehealth		checkbox	A general neurologist	1, Appointment by phone | 2, Appointment by video | 3, Appointment in person | 4, No appointment with this health professional | 888, Prefer not to answer		y
tele_doc_pt	veterans_survey_telehealth		checkbox	A physical therapist	1, Appointment by phone | 2, Appointment by video | 3, Appointment in person | 4, No appointment with this health professional | 888, Prefer not to answer		y
tele_doc_ot	veterans_survey_telehealth		checkbox	An occupational therapist	1, Appointment by phone | 2, Appointment by video | 3, Appointment in person | 4, No appointment with this health professional | 888, Prefer not to answer		y
tele_doc_slp	veterans_survey_telehealth		checkbox	A speech-language pathologist	1, Appointment by phone | 2, Appointment by video | 3, Appointment in person | 4, No appointment with this health professional | 888, Prefer not to answer		y
tele_doc_mh	veterans_survey_telehealth		checkbox	A mental health professional	1, Appointment by phone | 2, Appointment by video | 3, Appointment in person | 4, No appointment with this health professional | 888, Prefer not to answer		y
tele_doc_other	veterans_survey_telehealth		checkbox	Another doctor or medical provider	1, Appointment by phone | 2, Appointment by video | 3, Appointment in person | 4, No appointment with this health professional | 888, Prefer not to answer		y

tele_instructions	veterans_survey_telehealth		radio	Did your doctor's office provide you with instructions or technical support on how to use telehealth or virtual services for appointments? Instructions can include written or electronic directions about accessing the telehealth platform for your virtual appointment. Technical support is over-the-phone guidance provided by medical staff or an IT team and can include resetting a password or username or walking through how to download software to access a virtual appointment.	1, I was provided instructions | 2, I was provided technical support | 3, I was provided both technical support and instructions | 4, I was not provided with either technical support or instructions | 888, Prefer not to answer	[tele_use] = '1'	y
tele_support_open	veterans_survey_telehealth		notes	Optional: What type of support has been most helpful for learning how to use telehealth or virtual services for your appointments?		[tele_use] = '1'	
tele_satisfaction_pcp	veterans_survey_telehealth	How satisfied were you with these telehealth or virtual services compared to the same care you receive in person?	radio	A primary care or family doctor or provider	1, More satisfied with telehealth than in-person | 2, Equally satisfied with telehealth as in-person | 3, Less satisfied with telehealth than in-person | 4, Not sure | 5, Not Applicable, I did not use this service via telehealth | 888, Prefer not to answer	[tele_doc_pcp(2)] = '1' or [tele_doc_pcp(1)] = '1'	y
tele_satisfaction_mds	veterans_survey_telehealth		radio	A neurologist who specializes in Parkinson's (movement disorder specialist)	1, More satisfied with telehealth than in-person | 2, Equally satisfied with telehealth as in-person | 3, Less satisfied with telehealth than in-person | 4, Not sure | 5, Not Applicable, I did not use this service via telehealth | 888, Prefer not to answer	[tele_doc_mds(2)] = '1' or [tele_doc_mds(1)] = '1'	y
tele_satisfaction_neuro	veterans_survey_telehealth		radio	A general neurologist	1, More satisfied with telehealth than in-person | 2, Equally satisfied with telehealth as in-person | 3, Less satisfied with telehealth than in-person | 4, Not sure | 5, Not Applicable, I did not use this service via telehealth | 888, Prefer not to answer	[tele_doc_neuro(2)] = '1' or [tele_doc_neuro(1)] = '1'	y

tele_satisfaction_pt	veterans_survey_telehealth		radio	A physical therapist	1, More satisfied with telehealth than in-person | 2, Equally satisfied with telehealth as in-person | 3, Less satisfied with telehealth than in-person | 4, Not sure | 5, Not Applicable, I did not use this service via telehealth | 888, Prefer not to answer	[tele_doc_pt(2)] = '1' or [tele_doc_pt(1)] = '1'	y
tele_satisfaction_ot	veterans_survey_telehealth		radio	An occupational therapist	1, More satisfied with telehealth than in-person | 2, Equally satisfied with telehealth as in-person | 3, Less satisfied with telehealth than in-person | 4, Not sure | 5, Not Applicable, I did not use this service via telehealth | 888, Prefer not to answer	[tele_doc_ot(2)] = '1' or [tele_doc_ot(1)] = '1'	y
tele_satisfaction_slp	veterans_survey_telehealth		radio	A speech-language pathologist	1, More satisfied with telehealth than in-person | 2, Equally satisfied with telehealth as in-person | 3, Less satisfied with telehealth than in-person | 4, Not sure | 5, Not Applicable, I did not use this service via telehealth | 888, Prefer not to answer	[tele_doc_slp(2)] = '1' or [tele_doc_slp(1)] = '1'	y
tele_satisfaction_mh	veterans_survey_telehealth		radio	A mental health professional	1, More satisfied with telehealth than in-person | 2, Equally satisfied with telehealth as in-person | 3, Less satisfied with telehealth than in-person | 4, Not sure | 5, Not Applicable, I did not use this service via telehealth | 888, Prefer not to answer	[tele_doc_mh(2)] = '1' or [tele_doc_mh(1)] = '1'	y
tele_satisfaction_other	veterans_survey_telehealth		radio	Another doctor or medical provider	1, More satisfied with telehealth than in-person | 2, Equally satisfied with telehealth as in-person | 3, Less satisfied with telehealth than in-person | 4, Not sure | 5, Not Applicable, I did not use this service via telehealth | 888, Prefer not to answer	[tele_doc_other(2)] = '1' or [tele_doc_other(1)] = '1'	y

cpinv_cp	veterans_survey_care_partner_involvement	The next few questions will ask you about the assistance you receive from your care partner or a home health assistant. Please respond to the questions from your perspective.	radio	Do you have a care partner or caregiver who assists you on a regular basis?	1, Yes | 2, No | 888, Prefer not to answer		y
cpinv_relation	veterans_survey_care_partner_involvement		radio	What relation is your care partner to you? They are my...	1, Spouse or partner | 2, Parent | 3, Sibling | 4, Child | 5, Grandchild | 6, Friend | 7, Paid medical assistant | 8, Other | 888, Prefer not to answer	[cpinv_cp] = '1'	y
cpinv_cannot	veterans_survey_care_partner_involvement		radio	Are there things that you would like assistance with that your care partner cannot help you? This can include care partner physical limitations, illnesses or work schedules.	1, Yes | 2, No | 888, Prefer not to answer	[cpinv_cp] = '1'	y
cpinv_want	veterans_survey_care_partner_involvement		radio	Are there things you would like assistance with that you have not or do not want to ask from your care partner?	1, Yes | 2, No | 888, Prefer not to answer	[cpinv_cp] = '1'	y
cpinv_appts	veterans_survey_care_partner_involvement		radio	How often does your care partner attend your Parkinson's related medical or therapy appointments?	1, Always | 2, Sometimes | 3, Rarely | 4, Never | 888, Prefer not to answer	[cpinv_cp] = '1'	y
cpinv_conf_meds	veterans_survey_care_partner_involvement	How confident are you that your care partner knows about the following...	radio	The medications you take	1, Very confident | 2, Somewhat confident | 3, Slightly confident | 4, Not at all confident | 5, Not applicable | 888, Prefer not to answer	[cpinv_cp] = '1'	y
cpinv_conf_med_schedule	veterans_survey_care_partner_involvement		radio	Your medication schedule	1, Very confident | 2, Somewhat confident | 3, Slightly confident | 4, Not at all confident | 5, Not applicable | 888, Prefer not to answer	[cpinv_cp] = '1'	y
cpinv_conf_docs	veterans_survey_care_partner_involvement		radio	The medical doctors you see	1, Very confident | 2, Somewhat confident | 3, Slightly confident | 4, Not at all confident | 5, Not applicable | 888, Prefer not to answer	[cpinv_cp] = '1'	y

cpinv_conf_allied_health	veterans_survey_care_partner_involvement		radio	The allied health professionals you see (e.g., physical therapist, occupational therapist, mental health professional)	1, Very confident | 2, Somewhat confident | 3, Slightly confident | 4, Not at all confident | 5, Not applicable | 888, Prefer not to answer	[cpinv_cp] = '1'	y
cpinv_conf_va	veterans_survey_care_partner_involvement		radio	Benefits or resources at the Veterans Administration	1, Very confident | 2, Somewhat confident | 3, Slightly confident | 4, Not at all confident | 5, Not applicable | 888, Prefer not to answer	[cpinv_cp] = '1'	y
cpinv_conf_pdorgs	veterans_survey_care_partner_involvement		radio	Resources at local or national Parkinson's organizations	1, Very confident | 2, Somewhat confident | 3, Slightly confident | 4, Not at all confident | 5, Not applicable | 888, Prefer not to answer	[cpinv_cp] = '1'	y
cpinv_rely	veterans_survey_care_partner_involvement		radio	For what do you most rely on your care partner?	1, Assistance with household chores and errands | 2, Assistance with hygiene and getting dressed | 3, Assistance with moving around (e.g., transferring in and out of bed) | 4, Assistance with medications and treatments (including physical or occupational therapy) | 5, Transportation | 6, Scheduling medical appointments | 7, Communication (with medical professionals or family) | 8, Emotional support | 888, Prefer not to answer	[cpinv_cp] = '1'	y
anything_else	veterans_survey_last_thoughts		notes	Optional: As a person with Parkinson's, is there anything else you would like to tell us about your Parkinson's experience?			
recontact_falls	veterans_survey_last_thoughts	There are a few more questions that we would like to ask you. However, we want be respectful of your time. The first is a 10 question survey about falls and the second is 15 question survey is about mental health.	radio	Would you be interested in completing the additional falls survey next week?	1, Yes | 2, No		y

recontact_mh	veterans_survey_last_thoughts		radio	Would you be interested in completing the additional mental health survey next week?	1, Yes | 2, No		y
recontact_first	veterans_survey_last_thoughts		text	Please provide your first name so that we can recontact you:		[[recontact_falls] = '1' or [recontact_mh] = '1'	y
recontact_last	veterans_survey_last_thoughts		text	Please provide your last name so that we can recontact you:		[[recontact_falls] = '1' or [recontact_mh] = '1'	y
recontact_email	veterans_survey_last_thoughts		text	Please provide your email so that we can recontact you:		[recontact_falls] = '1' or [recontact_mh] = '1'	y
fes_1	followup_veterans_survey_falls	On a scale from 1 to 10, with 1 being very confident and 10 being not confident at all, how confident are you that you do the following activities without falling?	dropdown	Take a bath or shower	1, 1 (Very confident) | 2, 2 | 3, 3 | 4, 4 | 5, 5 | 6, 6 | 7, 7 | 8, 8 | 9, 9 | 10, 10 (Not at all confident)		y
fes_2	followup_veterans_survey_falls		dropdown	Reach into cabinets or closets	1, 1 (Very confident) | 2, 2 | 3, 3 | 4, 4 | 5, 5 | 6, 6 | 7, 7 | 8, 8 | 9, 9 | 10, 10 (Not at all confident)		y
fes_3	followup_veterans_survey_falls		dropdown	Walk around the house	1, 1 (Very confident) | 2, 2 | 3, 3 | 4, 4 | 5, 5 | 6, 6 | 7, 7 | 8, 8 | 9, 9 | 10, 10 (Not at all confident)		y
fes_4	followup_veterans_survey_falls		dropdown	Prepare meals not requiring carrying heavy or hot objects	1, 1 (Very confident) | 2, 2 | 3, 3 | 4, 4 | 5, 5 | 6, 6 | 7, 7 | 8, 8 | 9, 9 | 10, 10 (Not at all confident)		y
fes_5	followup_veterans_survey_falls		dropdown	Get in and out of bed	1, 1 (Very confident) | 2, 2 | 3, 3 | 4, 4 | 5, 5 | 6, 6 | 7, 7 | 8, 8 | 9, 9 | 10, 10 (Not at all confident)		y
fes_6	followup_veterans_survey_falls		dropdown	Answer the door or telephone	1, 1 (Very confident) | 2, 2 | 3, 3 | 4, 4 | 5, 5 | 6, 6 | 7, 7 | 8, 8 | 9, 9 | 10, 10 (Not at all confident)		y
fes_7	followup_veterans_survey_falls		dropdown	Get in and out of a chair	1, 1 (Very confident) | 2, 2 | 3, 3 | 4, 4 | 5, 5 | 6, 6 | 7, 7 | 8, 8 | 9, 9 | 10, 10 (Not at all confident)		y
fes_8	followup_veterans_survey_falls		dropdown	Getting dressed and undressed	1, 1 (Very confident) | 2, 2 | 3, 3 | 4, 4 | 5, 5 | 6, 6 | 7, 7 | 8, 8 | 9, 9 | 10, 10 (Not at all confident)		y

fes_9	followup_veterans_survey_falls		dropdown	Personal grooming (i.e., washing your face)	1, 1 (Very confident) | 2, 2 | 3, 3 | 4, 4 | 5, 5 | 6, 6 | 7, 7 | 8, 8 | 9, 9 | 10, 10 (Not at all confident)		y
fes_10	followup_veterans_survey_falls		dropdown	Getting on and off of the toilet	1, 1 (Very confident) | 2, 2 | 3, 3 | 4, 4 | 5, 5 | 6, 6 | 7, 7 | 8, 8 | 9, 9 | 10, 10 (Not at all confident)		y
gds_1	followup_veterans_survey_mental_health	Choose the best answer for how you have felt over the past week:	radio	Are you basically satisfied with your life?	0, Yes | 1, No		y
gds_2	followup_veterans_survey_mental_health		radio	Have you dropped many of your activities and interests?	1, Yes | 0, No		y
gds_3	followup_veterans_survey_mental_health		radio	Do you feel that your life is empty?	1, Yes | 0, No		y
gds_4	followup_veterans_survey_mental_health		radio	Do you often get bored?	1, Yes | 0, No		y
gds_5	followup_veterans_survey_mental_health		radio	Are you in good spirits most of the time?	0, Yes | 1, No		y
gds_6	followup_veterans_survey_mental_health		radio	Are you afraid that something bad is going to happen to you?	1, Yes | 0, No		y
gds_7	followup_veterans_survey_mental_health		radio	Do you feel happy most of the time?	0, Yes | 1, No		y
gds_8	followup_veterans_survey_mental_health		radio	Do you often feel helpless?	1, Yes | 0, No		y
gds_9	followup_veterans_survey_mental_health		radio	Do you prefer to stay at home, rather than going out and doing new things?	1, Yes | 0, No		y
gds_10	followup_veterans_survey_mental_health		radio	Do you feel you have more problems with memory than most?	1, Yes | 0, No		y
gds_11	followup_veterans_survey_mental_health		radio	Do you think it is wonderful to be alive now?	0, Yes | 1, No		y
gds_12	followup_veterans_survey_mental_health		radio	Do you feel pretty worthless the way you are now?	1, Yes | 0, No		y

gds_13	followup_veterans_survey_mental_health		radio	Do you feel full of energy?	0, Yes | 1, No		y
gds_14	followup_veterans_survey_mental_health		radio	Do you feel that your situation is hopeless?	1, Yes | 0, No		y
gds_15	followup_veterans_survey_mental_health		radio	Do you think that most people are better off than you are?	1, Yes | 0, No		y
