# Supplementary material for: Understanding health care needs among Veterans with Parkinson's disease: A survey study
Source: Front Neurol. 2022 Aug 11;13:924999. doi: 10.3389/fneur.2022.924999 (PMC9405651; doi:10.3389/fneur.2022.924999)

Supplementary Material

Supplemental Table 1: Fall and Near Fall Frequency (n = 366)

|  | **Fall** | **Near Fall** |
| --- | --- | --- |
| **Daily** | 8  (2.2%) | 50  (13.7%) |
| **Weekly** | 33  (9.0%) | 67  (18.3%) |
| **Monthly** | 37  (10.1%) | 36  (9.8%) |
| **Rarely** | 67  (18.3%) | 123  (33.6%) |
| **Not at All** | 217  (59.3%) | 86  (23.5%) |
| **Prefer Not to Respond** | 4  (1.1%) | 4  (1.1%) |

Supplemental Figure 1: Data Cleaning Flow Chart


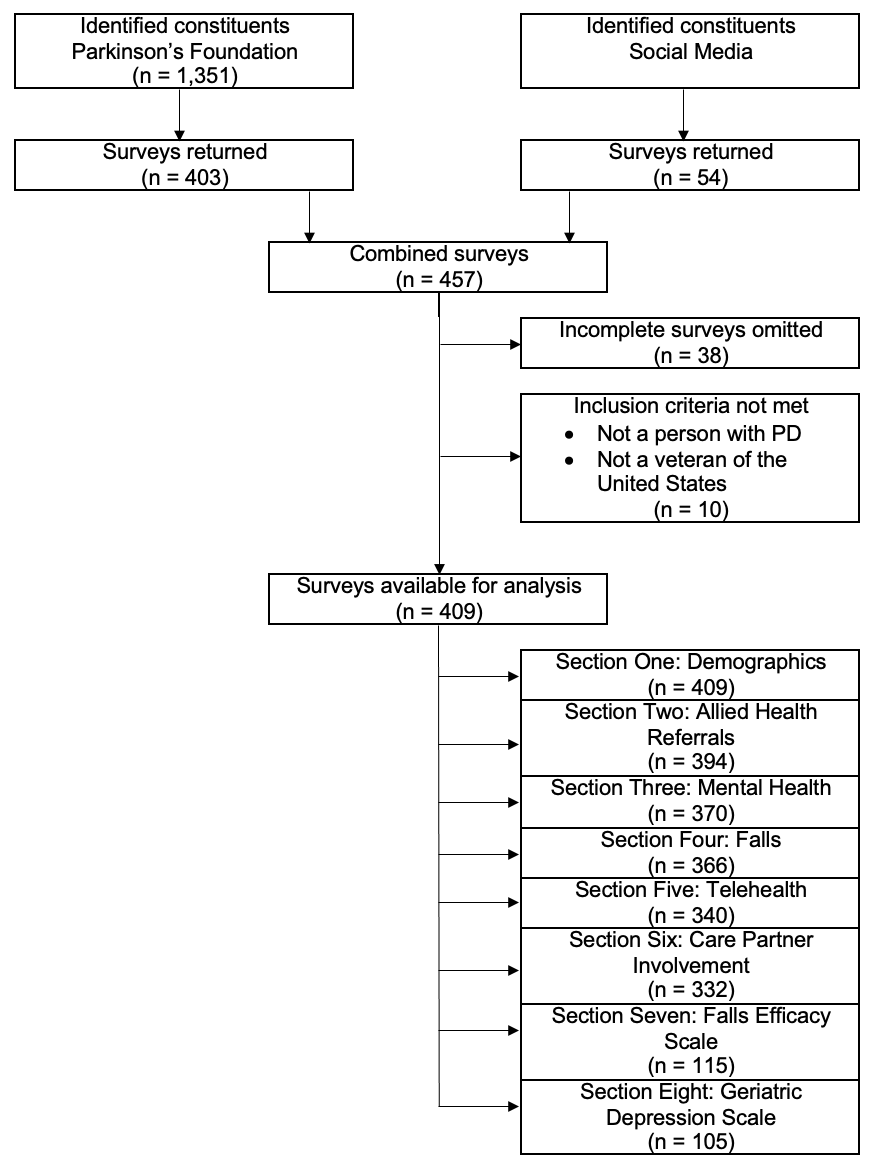

Supplement: Supplementary file 2 [file Data_Sheet_2.docx]
